# Notch signaling promotes nephrogenesis by downregulating Six2

**DOI:** 10.1242/dev.143503

**Published:** 2016-11-01

**Authors:** Eunah Chung, Patrick Deacon, Sierra Marable, Juhyun Shin, Joo-Seop Park

**Affiliations:** Division of Pediatric Urology and Division of Developmental Biology, Cincinnati Children's Hospital Medical Center, 3333 Burnet Avenue, Cincinnati, OH 45229, USA

**Keywords:** Notch, Six2, Nephron progenitors, Nephrogenesis, Kidney, Nephron segmentation, Mouse

## Abstract

During nephrogenesis, multipotent mesenchymal nephron progenitors develop into distinct epithelial segments. Each nephron segment has distinct cell types and physiological function. In the current model of kidney development, Notch signaling promotes the formation of proximal tubules and represses the formation of distal tubules. Here, we present a novel role of Notch in nephrogenesis. We show in mice that differentiation of nephron progenitors requires downregulation of Six2, a transcription factor required for progenitor maintenance, and that Notch signaling is necessary and sufficient for Six2 downregulation. Furthermore, we find that nephron progenitors lacking Notch signaling fail to differentiate into any nephron segments, not just proximal tubules. Our results demonstrate how cell fates of progenitors are regulated by a transcription factor governing progenitor status and by a differentiation signal in nephrogenesis.

## Abstract (precis)

**Summary:** Notch is necessary and sufficient for downregulation of Six2 during nephrogenesis, while nephron progenitors that lack Notch signaling fail to form all segments of the nephron, not just proximal tubules.

## INTRODUCTION

In mammals, nephrons are formed only during development, with nephrogenesis stopping at 36 weeks of gestation in humans and by the fourth postnatal day in mice (Hartman et al., 2007; Hinchliffe et al., 1991; Rumballe et al., 2011). Nephron progenitors residing at the cortex of the developing kidney undergo mesenchymal-to-epithelial transition (MET) and give rise to all of the epithelial cells of the nephron (McMahon, 2016). In order to generate a sufficient number of nephrons, it is crucial to balance the self-renewal and differentiation of nephron progenitors before they are depleted around birth.

Undifferentiated nephron progenitors express Six2, a homeobox transcription factor that gradually declines in expression as these progenitors undergo differentiation (Kobayashi et al., 2008; Park et al., 2012; Self et al., 2006). Deletion of *Six2* disrupts the balance between self-renewal and differentiation of nephron progenitors, resulting in premature depletion of nephron progenitors accompanied by ectopic nephrogenesis (Kobayashi et al., 2008; Self et al., 2006). This illustrates the pivotal role of Six2 in the maintenance of progenitors and implies that downregulation of Six2 is a critical step for the differentiation of nephron progenitors.

In addition to Six2, the Wnt/β-catenin and Notch signaling pathways are known to regulate cell fate decisions during nephrogenesis (McMahon, 2016). Activation of Wnt/β-catenin signaling blocks degradation of β-catenin, allowing β-catenin to regulate the expression of its target genes in the nucleus (Clevers and Nusse, 2012). Wnt/β-catenin is required for both the self-renewal and differentiation of nephron progenitors (Carroll et al., 2005; Karner et al., 2011; Park et al., 2007). During differentiation, Wnt/β-catenin signaling activates the expression of key differentiation genes, such as *Fgf8* and *Wnt4*, which are required for nephrogenesis (Grieshammer et al., 2005; Park et al., 2012, 2007; Perantoni et al., 2005; Stark et al., 1994). Notch signaling is believed to act downstream of Wnt/β-catenin signaling (Boyle et al., 2011; Park et al., 2012). Activation of Notch signaling leads to release of the intracellular domain (ICD) of the Notch receptor from the membrane. Notch ICD forms a complex with its DNA-binding partner Rbpj to regulate the expression of its target genes (Park and Kopan, 2015). In the current model of nephrogenesis, it is thought that Notch signaling is dispensable for the initiation of nephrogenesis and that it promotes the formation of the proximal segment of the nephron and represses the formation of the distal segment (Cheng et al., 2007, 2003; Park and Kopan, 2015).

Here we show definitively that downregulation of Six2 is required for the differentiation of nephron progenitors and that Notch is necessary and sufficient for downregulating Six2. Furthermore, our lineage analysis shows that Notch signaling is required for the formation of all segments of the nephron. This work reveals a novel role of Notch signaling in nephrogenesis.

## RESULTS

### Downregulation of Six2 is essential for the differentiation of nephron progenitors

**Fig. 1.**
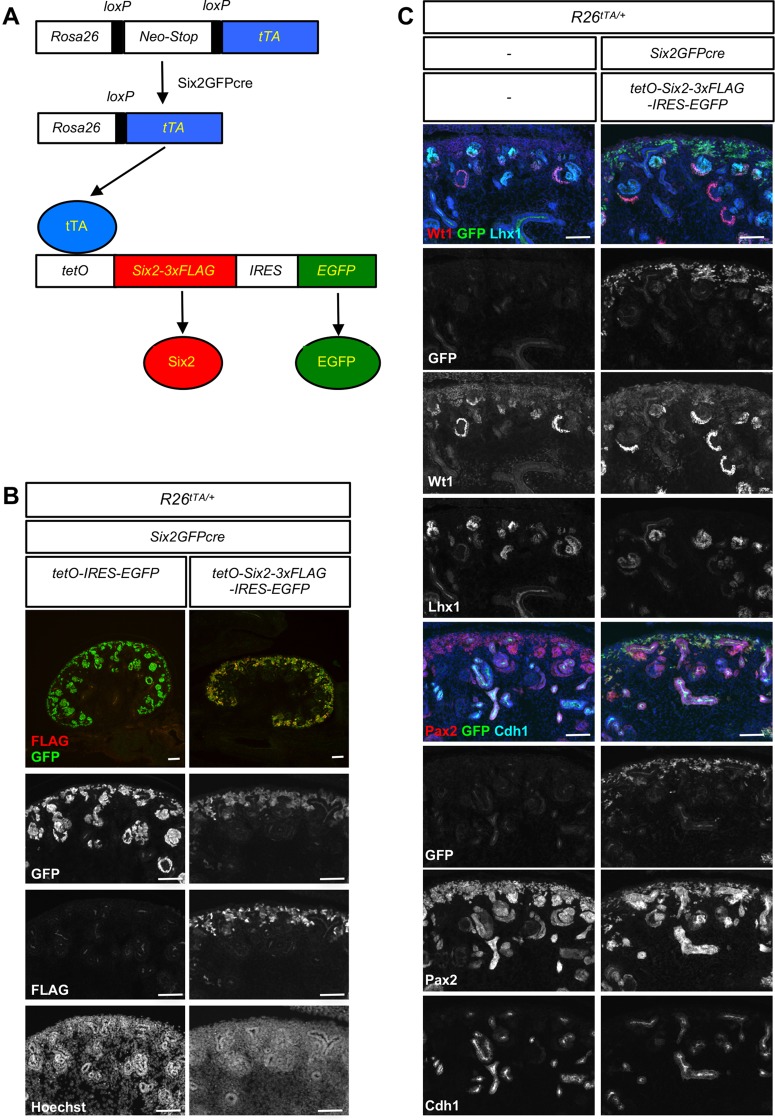
**Downregulation of Six2 is required for the differentiation of nephron progenitors.** (A) Generation of *Six2* gain-of-function (GOF) mutant. We generated a transgenic mouse line carrying *tetO*-regulated *Six2* followed by *IRES-EGFP*. When combined with *Rosa26-LNL-tTA* and *Six2GFPcre*, it expresses 3xFLAG-tagged Six2 and EGFP permanently in nephron progenitors and their descendants. (B) Constitutive expression of *Six2* restricts nephron progenitors to the cap mesenchyme. IRES-GFP serves as a lineage tracer and FLAG expression indicates expression of Six2 from the *tetO-Six2* transgene. In the control kidney (left column), GFP is expressed in the entire nephron lineage. In the *Six2* GOF mutant kidney (right column), GFP^+^ cells are restricted to the cap mesenchyme. (C) The *Six2* GOF mutant cells fail to differentiate. *Six2* GOF mutant cells (GFP^+^) fail to express the differentiation marker Lhx1 or the epithelial marker Cdh1 (E-cadherin), whereas they still express Wt1 and Pax2 in the cap mesenchyme. Embryonic kidneys at E14.5 were examined. Images are representative of two independent experiments. Scale bars: 100 µm.

In order to test whether downregulation of Six2 is required for the differentiation of nephron progenitors, we performed a *Six2* gain-of-function (GOF) study. We generated a transgenic mouse line carrying 3xFLAG-tagged Six2 and IRES-EGFP under the control of the tetracycline operator promoter (*tetO-Six2-IRES-EGFP*). We used a nephron lineage-specific Six2GFPcre to activate *Rosa26-LNL-tTA*, a floxed transcription stop cassette followed by a tetracycline-controlled (tet-off) transactivator (Kobayashi et al., 2008; Park et al., 2007; Wang et al., 2008). The combination of these three transgenes results in the expression of both FLAG-tagged Six2 and EGFP in the nephron lineage in the absence of tetracycline (Fig. 1A). Since this transgenic expression of Six2 from the *tetO* transgene is persistent even after endogenous expression of Six2 is turned off, FLAG or EGFP can serve as a lineage tracer. We confirmed that 3xFLAG-tagged Six2 is functionally equivalent to untagged Six2 in both transcriptional activation and repression (Fig. S1).

In the control kidney, GFP^+^ cells were found in developing nephrons (Fig. 1B, left column). By contrast, in the *Six2* GOF mutant kidney, GFP^+^ or FLAG^+^ cells were restricted to the cap mesenchyme, failing to escape from the progenitor niche (Fig. 1B, right column). The absence of GFP^+^ nephrons suggests that nephron progenitors constitutively expressing *Six2* do not go on to form nephrons. A more detailed analysis of the *Six2* GOF mutant kidney showed that, similar to the control kidney, the mutant nephron progenitors in the cap mesenchyme expressed Wt1 and Pax2, two key transcription factors required for proper nephrogenesis (Fig. 1C). Six2 and proliferation marker expression in the *Six2* GOF mutant kidney were also similar to those of the control kidney (Fig. S2). However, the mutant nephron progenitors failed to differentiate into Lhx1^+^ nephron tubules or Cdh1^+^ epithelial cells, as shown by the lack of GFP lineage tracer in these cell types (Fig. 1C, right panels). This demonstrates that persistent expression, rather than overexpression, of Six2 prevents mesenchymal nephron progenitors from differentiating into epithelial nephron tubules, strongly suggesting that downregulation of Six2 is required for nephrogenesis. Interestingly, some nephron tubules do form in the *Six2* GOF mutant kidney (Fig. 1C). However, these nephron tubules were negative for both FLAG and GFP, indicating that these cells were descendants of progenitors that escaped Six2GFPcre-mediated recombination and failed to express transgenic *Six2*. This was likely to have been caused by mosaic expression of Six2GFPcre (Surendran et al., 2010).

### Notch signaling is necessary and sufficient for downregulation of Six2

**Fig. 2.**
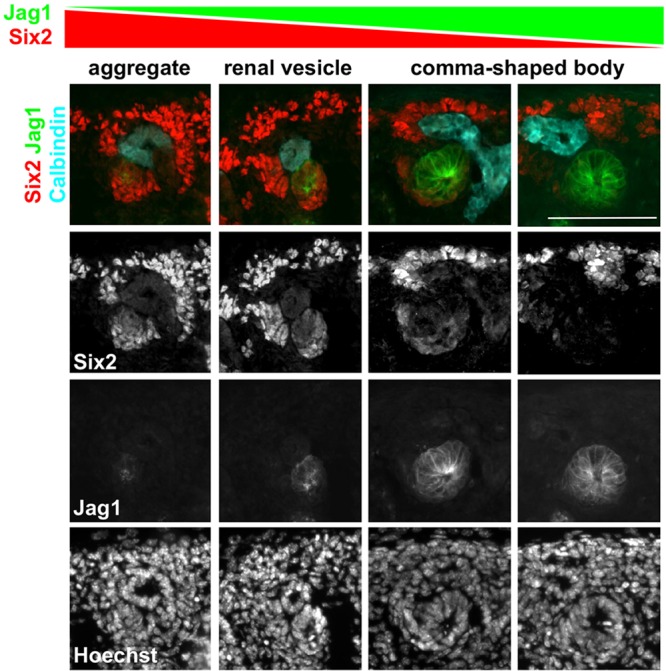
**Inverse correlation of Jag1 and Six2 expression.** Expression of Jag1 in the aggregate and the renal vesicle coincides with the downregulation of Six2. As the expression domain of Jag1 expands in the comma-shaped body, little or no Six2 is detected. E16.5 kidneys are shown. Images are representative of two independent experiments. Scale bar: 100 µm.

Combined with previous *Six2* loss-of-function (LOF) studies (Kobayashi et al., 2008; Self et al., 2006), our *Six2* GOF analysis suggests that expression of Six2 needs to be tightly regulated in order to balance the self-renewal and differentiation of nephron progenitors. Six2 expression is gradually lost during the differentiation of nephron progenitors (Park et al., 2012). We observed that downregulation of Six2 coincided with the expression of Jag1, the major Notch ligand in the process of nephrogenesis (Fig. 2). Expression of Jag1 was polarized in the developing nephron, as previously reported (Cheng et al., 2007; Georgas et al., 2009; Park et al., 2012). In the aggregate and renal vesicle (RV), Jag1 was detected in the distal part that is adjacent to the tip of the collecting duct. At the same time, downregulation of Six2 also occurred at the same distal part of the aggregate and RV. Six2 was completely lost when the expression domain of Jag1 expanded to the entire comma-shaped body (Fig. 2). This result showed an inverse correlation of Jag1 and Six2 expression and raised the possibility that Notch signaling downregulates Six2 in aggregates and RVs.

**Fig. 3.**
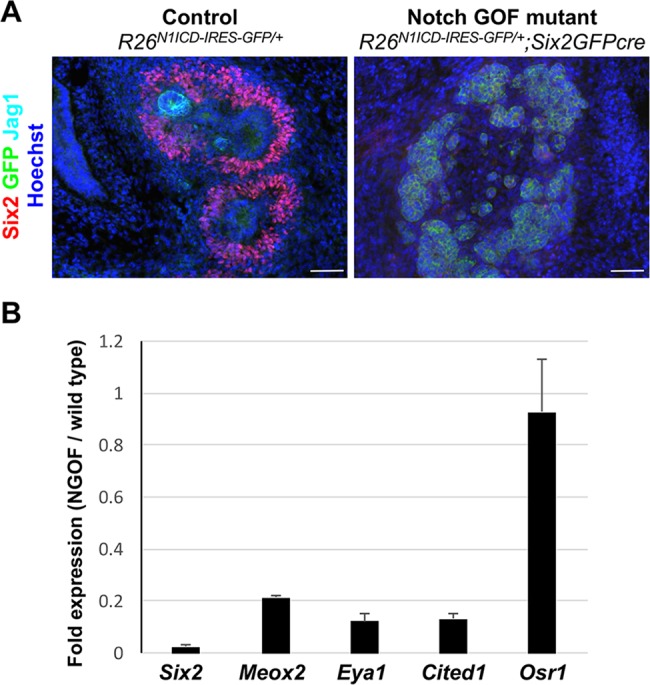
**Notch signaling is sufficient for downregulation of Six2.** (A) Expression of an active form of Notch in nephron progenitors depletes Six2^+^ cells. Scale bars: 100 µm. (B) RT-qPCR analysis showing that all cap mesenchyme markers are downregulated in the Notch GOF mutant kidney, except for *Osr1*. E13.5 kidneys are shown. Results are representative of two independent experiments. *n*=2; error bars indicate s.d.

To investigate whether Notch signaling regulates the expression of Six2, we performed Notch GOF and LOF studies by utilizing Six2GFPcre to specifically target nephron progenitors and their descendants (Kobayashi et al., 2008; Park et al., 2007). We found that expression of an active form of Notch1 (Notch1 ICD) in nephron progenitors completely abolished the expression of Six2 (Fig. 3A). Our RT-qPCR analysis of several specific cap mesenchyme markers showed that *Osr1* was still expressed in the Notch GOF mutant kidney, whereas other markers, including *Six2*, were downregulated (Fig. 3B). This suggests that downregulation of Six2 in the Notch GOF mutant kidney is not simply due to depletion of nephron progenitors.

**Fig. 4.**
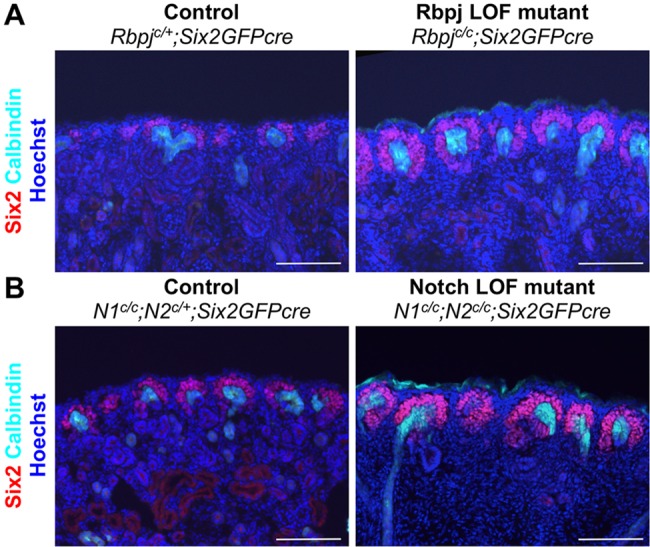
**Notch signaling is required for downregulation of Six2.** Deletion of *Rbpj* (A) or Notch receptors (B) causes expansion of Six2^+^ cells. Notably, in these mutants, Six2^+^ cells are found deeper into the medullary side of tips of the collecting duct because downregulation of Six2 is defective. P0 kidneys are shown. Images are representative of three independent experiments. Scale bars: 100 µm.

We found that deletion of either *Rbpj*, which is required for Notch signaling, or of two Notch receptor genes, namely *Notch1* and *Notch2*, caused a defect in the downregulation of Six2 (Fig. 4). In these mutants, Six2^+^ cells were found deeper at the medullary side of the tips of the collecting duct, most likely due to a lack of Six2 downregulation. Despite the apparent defect in Six2 downregulation, some nephron tubules appeared to form in these mutants, albeit at reduced frequency. This is caused, at least in part, by mosaic expression of Six2GFPcre. We found that the Lhx1^+^ nephron tubules formed in these mutant kidneys were often not labeled with lineage tracer (Fig. S3), suggesting that intact Notch signaling allowed the formation of Lhx1^+^ nephron tubules. Taken together, our Notch LOF and GOF data show that Notch signaling is necessary and sufficient for the downregulation of Six2.

### Nephron progenitors lacking Notch signaling fail to differentiate into any nephron segments

**Fig. 5.**
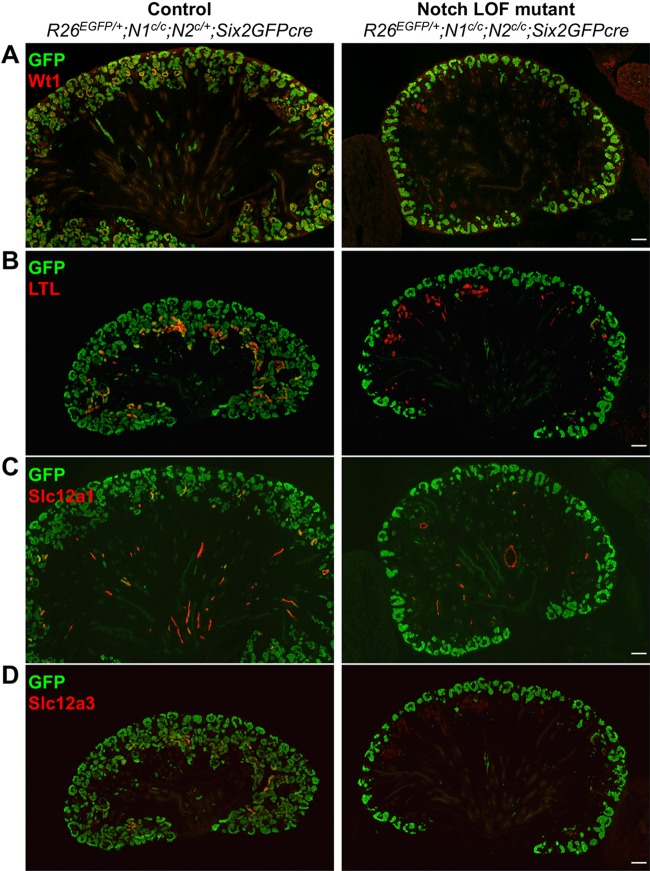
**Nephron progenitors lacking Notch receptors fail to form any nephron segments.** Lineage analysis of Six2^+^ cells shows that nephrogenesis is blocked in the *Notch1* and *Notch2* double-mutant kidney. In the control kidney (left column), RosaGFP reporter-positive cells form Wt1^+^ podocytes (A), LTL^+^ proximal tubules (B), Slc12a1^+^ loop of Henle (C) and Slc12a3^+^ distal tubules (D). In the Notch double-mutant kidney (right column), GFP^+^ cells fail to develop into any segment of the nephron. P0 kidneys are shown. Images are representative of two independent experiments. Scale bars: 100 µm.

Thus far, we have demonstrated that downregulation of Six2 is required for the formation of nephron tubules and that Notch signaling is necessary and sufficient for this downregulation. These results suggest that Notch signaling is required for the formation of all nephron segments. This is inconsistent with the current model of nephrogenesis, whereby Notch signaling promotes the formation of proximal tubules and podocytes but represses the formation of distal tubules (Cheng et al., 2007, 2003; Park and Kopan, 2015). To address this discrepancy, we examined which segments of the nephron could be formed from nephron progenitors lacking the Notch1 and Notch2 receptors by lineage analysis. We used Wt1 and *Lotus tetragonolobus* lectin (LTL) to mark podocytes and proximal tubules, respectively. In addition, we used Slc12a1 and Slc12a3 to label loop of Henle and distal tubules, respectively, because they have been reported to be specifically expressed in those segments (Lee et al., 2015). In the control kidneys, cells originating from RosaGFP reporter-labeled nephron progenitors formed podocytes (Wt1^+^), proximal tubules (LTL^+^), loop of Henle (Slc12a1^+^) and distal tubules (Slc12a3^+^) (Fig. 5, left panels). By contrast, in the Notch double-mutant kidneys, the RosaGFP reporter-labeled nephron progenitors failed to form any segments of the nephron (Fig. 5, right panels). Although each segment of the nephron was present in the Notch double-mutant kidneys, they were significantly reduced in number. Importantly, most of these cells were not labeled with the RosaGFP reporter, suggesting that they originated from progenitors that had escaped recombination by Six2GFPcre and thus expressed Notch genes. We also performed the same lineage analysis in the *Rbpj* LOF mutant kidneys, and found that the nephron progenitors differentiate poorly into any nephron segments (Fig. S4). Our data strongly suggest that Notch signaling is required for the formation of all segments of the nephron.

**Fig. 6.**
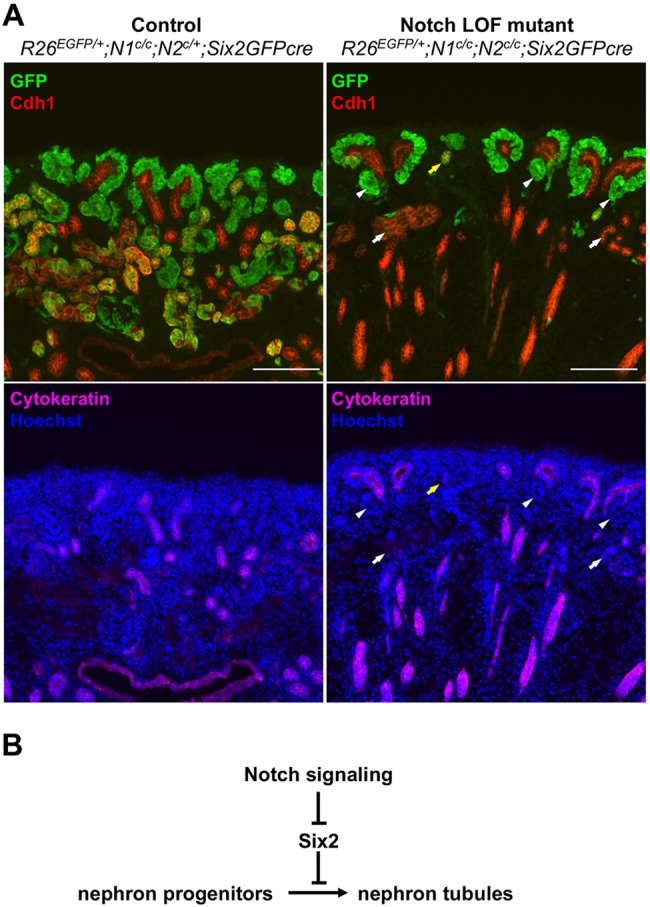
**Notch double-mutant kidneys form renal vesicles that fail to develop into Cdh1^+^ nephron tubules.** (A) Lineage analysis shows significant overlap of Cdh1 and GFP in the control kidney (left), indicating the formation of epithelial nephron tubules from nephron progenitors. In the Notch double-mutant kidney (right), Cdh1^+^ epithelial cells are either recombination escapers (EGFP^−^, white arrows) or collecting duct cells (cytokeratin^+^). The Notch double-mutant kidney forms renal vesicle-like structures that are Cdh1^−^ (white arrowheads). A rare GFP and Cdh1 double-positive structure in the Notch double-mutant kidney is marked with a yellow arrow. P0 kidneys are shown. Images are representative of three independent experiments. Scale bars: 100 µm. (B) Model of Notch-mediated regulation of nephrogenesis. Notch-mediated downregulation of Six2 is required for nephrogenesis. Since downregulation of Six2 occurs early in nephrogenesis, likely prior to nephron segmentation, Notch signaling is required for the formation of all segments of the nephron.

Our lineage analysis showed that the differentiation of nephron progenitors is severely defective in the absence of Notch signaling. This led us to examine whether Notch signaling is required for MET of nephron progenitors. In the control kidneys, we found significant overlap of Cdh1 (a pan-epithelial marker) and the RosaGFP reporter, indicating the expected MET of nephron progenitors (Fig. 6A, left panel). We also found that some nephron tubules (Cdh1^+^ cytokeratin^−^) were not marked with the RosaGFP reporter, showing again that expression of Six2GFPcre was mosaic. Interestingly, in the Notch double-mutant kidney, most of the RosaGFP reporter-labeled cells remained at the cortex of the kidney, unable to differentiate into Cdh1^+^ nephron tubules (Fig. 6A, right panel). The mutant kidneys formed some nephron tubules (Cdh1^+^ cytokeratin^−^) but most were not labeled with GFP (white arrows in Fig. 6A, right), suggesting that these nephron tubules arose from escapers of Cre recombination. Although most of the RosaGFP reporter-labeled cells in the Notch double-mutant kidney failed to differentiate into Cdh1^+^ nephron tubules, the mutant kidney did contain Cdh1^−^ RV-like structures (white arrowheads in Fig. 6A, right) but no S-shaped bodies (SSBs). It appeared that RVs did form in the Notch double-mutant kidney but they failed to develop into SSBs. Since RV is considered to be an epithelial structure, we conclude that Notch signaling is not required for MET of nephron progenitors, consistent with previous reports (Cheng et al., 2007, 2003; Wang et al., 2003).

## DISCUSSION

Notch signaling was previously thought to be dispensable for the early differentiation of nephron progenitors. Instead, it was believed to play a later role in nephron segmentation, with Notch required for formation of the proximal but not distal segment of the nephron (Cheng et al., 2007, 2003). Here we show that downregulation of Six2 is required for the differentiation of nephron progenitors and that Notch signaling is necessary and sufficient for Six2 downregulation. The finding that Notch is involved in the downregulation of Six2 suggests that Notch signaling has a profound impact on the gene regulatory network governing the maintenance of nephron progenitors. The Six2^+^ cell population expanded in the *Rbpj* or Notch LOF mutant kidneys (Fig. 4) and Six2 expression was abolished in the Notch GOF mutant kidney (Fig. 3). Our results showing that Notch-mediated downregulation of Six2 is required for nephrogenesis predicted that Notch signaling would be required for the formation of all nephron segments regardless of the different cell fates along the proximal-distal axis of the nephron. This prediction was inconsistent with the current model, whereby Notch signaling promotes the formation of the proximal segment and represses the formation of the distal segment of the nephron (Cheng et al., 2007, 2003). Strikingly, nephron progenitors lacking both *Notch1* and *Notch2* failed to form not only the proximal tubule but also the distal tubule, loop of Henle and podocytes (Fig. 5). We also found largely similar results in the *Rbpj* LOF mutant kidney. Unlike loss of Notch receptors, loss of Rbpj did not completely block nephron segmentation (Fig. S4). This might be due to the fact that, in the *Rbpj* LOF mutant kidney, a small minority of cells labeled with lineage tracer still express Rbpj (Fig. S5). We suspect that this was a result of either incomplete removal of floxed *Rbpj* or the persistence of Rbpj protein. Nonetheless, the *Rbpj* LOF mutant nephron progenitors differentiate poorly into any specific nephron segment. Our results strongly support a new model, in which Notch signaling promotes nephrogenesis by downregulating Six2 expression (Fig. 6B).

The previous model for the role of Notch signaling in nephrogenesis was, at least in part, based on the characterization of *Notch2* LOF mutant kidneys (Cheng et al., 2007). In that study, when *Notch2* was deleted with Pax3Cre, which targets both nephron and interstitium lineages in the kidney (Engleka et al., 2005), nephrogenesis was severely defective. However, in another study, when the nephron lineage-specific Six2GFPcre was used to delete *Notch2*, the mutant kidney showed no nephron segmentation defect, although it generated fewer nephrons (Surendran et al., 2010). Deletion of both *Notch1* and *Notch2* receptors with Six2GFPcre phenocopies the deletion of *Rbpj* with Six2GFPcre, causing a severe defect in nephrogenesis, which indicates that *Notch1* and *Notch2* act redundantly in the nephron lineage (Surendran et al., 2010). In order to address Notch function in nephrogenesis, we removed both *Notch1* and *Notch2* with the nephron-specific Six2GFPcre and performed extensive characterization of the mutant kidney with lineage analysis.

Our data show that the Notch double-mutant kidneys formed RV that fails to develop into SSB. This is consistent with previous reports demonstrating that pharmacological inhibition or genetic removal of γ-secretase activity allows the formation of RV but not SSB (Cheng et al., 2003; Wang et al., 2003). The notion that the distal tubules are still formed in the absence of Notch signaling was in part due to the use of Cdh1 (E-cadherin) as a distal tubule marker (Cheng et al., 2007, 2003). Although the distal and median segments of SSBs have higher expression of Cdh1 than the proximal segment (Barker et al., 2012; Cheng et al., 2007), most of the segments of the nephron tubules in the developing kidney express Cdh1, except for Bowman's capsule and podocytes (Fig. 6A). Thus, Cdh1 is not an adequate marker to distinguish proximal and distal tubules of the nephron. We observed that, in the Notch double-mutant kidney, a small number of Rosa reporter-positive cells did become Cdh1^+^ epithelial cells (Fig. 6A, yellow arrowhead). Unlike nephron tubules in the wild-type kidney, these cells are not elongated and do not express any nephron segmentation markers, suggesting that they failed to differentiate into mature nephron tubules. Despite the fact that RVs are formed in the Notch double-mutant kidney, the paucity of Cdh1^+^ nephron tubules in the mutant kidney suggests that Notch signaling is required for the differentiation of nephron progenitors (Fig. 6B).

Two Notch GOF studies have been reported using Six2GFPcre to activate the expression of Notch ICD, the active form of Notch, from the *Rosa26* locus. Expression of Notch1 ICD lacking the PEST domain caused the ectopic formation of proximal tubules (Cheng et al., 2007), while expression of the full-length Notch2 ICD with the PEST domain caused premature depletion of nephron progenitors without the ectopic formation of proximal tubules (Fujimura et al., 2010). It was also shown that, similar to the *Six2* LOF mutant kidney, the *Notch2* GOF mutant kidney ectopically expresses *Wnt4*, a key differentiation gene in nephrogenesis. Since the PEST domain is involved in the degradation of Notch (Chiang et al., 2006; Fryer et al., 2004), Notch ICD lacking the PEST domain is believed to be more stable, and hence more potent, than full-length Notch ICD (Murtaugh et al., 2003). It is unclear whether the ectopic formation of proximal tubules is due to the potency of the presumably more stable Notch ICD or whether the difference originates from the type of Notch receptor. Nonetheless, the premature depletion of nephron progenitors occurred in both Notch GOF mutants, consistent with our finding that Notch signaling downregulates Six2.

We found that constitutive expression of Six2 prevents nephron progenitors from differentiating into nephron tubules, suggesting that downregulation of Six2 is required for nephrogenesis. We have previously reported that Six2 and β-catenin share common transcriptional targets in nephron progenitors (Park et al., 2012). Among their common targets are *Wnt4* and *Fgf8*, both of which are required for nephrogenesis (Grieshammer et al., 2005; Perantoni et al., 2005; Stark et al., 1994) and are activated by β-catenin (Park et al., 2012, 2007). We previously showed that β-catenin forms a complex with Six2 *in vitro* and *in vivo* (Park et al., 2012). It is known that β-catenin is required for the differentiation of nephron progenitors (Park et al., 2007), and in this report we show that constitutive expression of Six2 can block nephrogenesis. These results suggest that Six2 might block the expression of β-catenin targets that drive the differentiation of nephron progenitors. This idea is supported by the fact that Six2 can repress β-catenin-mediated transcriptional activation (Fig. S1B). However, it has been shown that β-catenin is required for nephron progenitor maintenance (Karner et al., 2011). It remains to be determined how Six2 antagonizes β-catenin-mediated initiation of nephrogenesis but not β-catenin-mediated nephron progenitor maintenance.

The inverse correlation of Six2 and Jag1 expression during nephrogenesis (Fig. 2) and the Six2 expression patterns in the Notch LOF and GOF mutant kidneys (Figs 3 and 4) provide compelling evidence to support the idea that Notch signaling downregulates Six2. Consistent with this, it was recently shown that pharmacological inhibition of Notch signaling promotes self-renewal of Six2^+^ nephron progenitors *in vitro* (Tanigawa et al., 2016; Yuri et al., 2015). Expression of Six2 appears to be regulated by multiple factors. We have previously shown that an enhancer located 60 kb upstream of the *Six2* gene is capable of driving a transgenic reporter in Six2^+^ cells (Park et al., 2012). This enhancer was bound by both Six2 and β-catenin. Since treatment of nephron progenitors with a GSK inhibitor causes repression of Six2, we concluded that β-catenin contributes to the repression of Six2. However, it was recently reported that Wnt/β-catenin signaling is required for the maintenance of nephron progenitors *in vivo* and *in vitro* (Brown et al., 2015; Karner et al., 2011; Tanigawa et al., 2016). A low level of β-catenin might be required for the maintenance of Six2 expression and a high level of β-catenin might contribute to the downregulation of Six2. It is possible that Wnt/β-catenin and Notch signals coordinate to regulate Six2 expression.

Our findings have implications for cell replacement therapy of the kidney. Much effort has been focused on the *in vitro* generation of nephron tubules by directed differentiation of embryonic stem cells (Morizane et al., 2015; Takasato et al., 2015; Tanigawa et al., 2016). In most cases, Wnt/β-catenin signaling is employed to initiate the differentiation of nephron progenitors, even though Wnt/β-catenin signaling is also important for the maintenance of nephron progenitors (Brown et al., 2015; Karner et al., 2011). Our work suggests that Notch signaling may serve as a more efficient trigger for the differentiation of nephron progenitors into nephron tubules.

## MATERIALS AND METHODS

### Mouse strains

*tetO-Six2-IRES-EGFP* and *tetO-IRES-EGFP* transgenic lines were generated by pronuclear injection at the Cincinnati Children's Hospital Medical Center (CCHMC) Transgenic Animal and Genome Editing Core. Six2 was tagged with 3xFLAG at the C-terminus. Founders for each line were outcrossed to Swiss Webster. BAC transgenic *Six2GFPcre* (Kobayashi et al., 2008; Park et al., 2007), *Rosa26-Notch1ICD-IRES-GFP* (Murtaugh et al., 2003), *Rosa26-LNL-tTA* (Wang et al., 2008), *Rosa26-EGFP* (Srinivas et al., 2001), *Rbpj^c/c^* (Tanigaki et al., 2002), *Notch1^c/c^* (Yang et al., 2004) and *Notch2^c/c^* (McCright et al., 2006) mice were described previously. All mice were maintained in the CCHMC animal facility according to animal care regulations. The Animal Studies Committee of CCHMC approved the experimental protocols (IACUC2013-0105).

### Immunofluorescence

Embryonic or newborn (P0) kidneys were fixed with 4% PFA in PBS, incubated in 30% sucrose in PBS at 4°C overnight, and imbedded in OCT (Fisher Scientific). Cryosections (10 µm) were incubated overnight with 5% heat-inactivated sheep serum/PBST (PBS with 0.1% Triton X-100) containing primary antibodies. Antibodies are described in Table S1. Fluorophore-labeled secondary antibodies (Invitrogen or Jackson ImmunoResearch) were used for indirect visualization. Images were taken with a Zeiss ApoTome or Nikon Ti-E SpectraX widefield microscope.

### Reporter assays

*Six2* promoter-driven reporter and SuperTopFlash reporter assays were performed as described in the supplementary Materials and Methods.

### Real-time quantitative PCR (RT-qPCR)

Control or Notch GOF embryonic kidneys were dissected out and total RNA was extracted using the Qiagen RNeasy Micro Kit according to manufacturer's instructions for microdissected tissue. Using ∼1 µg total RNA, reverse transcription was performed using the RevertAid cDNA Synthesis Kit (Thermo Scientific, K1621) to obtain cDNA. qPCR was performed on an Applied Biosystems StepOne Plus (Thermo Scientific) using Power SYBR Green PCR Master Mix (Thermo Scientific, 4368706). Oligonucleotide primers (5′-3′, forward and reverse) used were: *Gapdh*, CAACTTTGTCAAGCTCATTTCCTG and CCTCTCTTGCTCAGTGTCCTT; *Six2*, ACATGAGGGCGTAAAATGGA and CACCTCGCTGGTT-CTTCTCT; *Meox2*, GTGCGGCAAATGTCTGATTT and GCTTTGTTTGGCACTTGGTT; *Eya1*, ATGGCAACACAAAGACCACA and AGGGTGATGGGAGAAACACA; *Cited1*, AGCAGCCAGAGGGAAAATCT and GGATGAGGAGGTGCTGATGT; *Osr1*, CCGGAAGGAAAACTGCATTA and CGGAGTTTTCGTTGTGTGTG. Two biological replicates each of control and Notch1 ICD-expressing E13.5 kidneys were used. Fold change calculations were performed using the ΔΔCt method.
